# Network pharmacology, bioinformatics and in vitro/in vivo validation elucidate the anti-lung cancer activities and potential targets of Rhoifolin

**DOI:** 10.3389/fphar.2025.1727729

**Published:** 2026-01-14

**Authors:** Jing Qian, Wei Cheng, Shuangyan Li, Li Deng, Di Gao, Xue Zhang, Yunhui Zhang

**Affiliations:** 1 Department of Respiratory Medicine, The First People’s Hospital of Yunnan Province, Kunming University of Science and Technology Affiliated Hospital, Kunming, China; 2 Chongqing Key Laboratory of New Drug Screening from Traditional Chinese Medicine, Integrative Science Center of Germplasm Creation in Western China (Chongqing) Science City and Southwest University, SWU-TAAHC Medicinal Plant Joint R&D Centre, College of Pharmaceutical Sciences, Southwest University, Chongqing, China; 3 Department of Oncology, 920th Hospital of the Joint Logistics Support Force of PLA, Kunming, China; 4 Department of Pathology, The First People’s Hospital of Yunnan Province, Kunming University of Science and Technology Affiliated Hospital, Kunming, China; 5 College of Foreign Languages, Qingdao City University, Qingdao, China

**Keywords:** EPHB2, lung cancer, molecular dynamics simulations, network pharmacology, Rhoifolin (ROF)

## Abstract

**Background:**

Rhoifolin (ROF), a naturally occurring flavonoid, exhibits broad bioactivities, but its therapeutic potential and underlying mechanisms in lung cancer remain largely unknown. This study was designed to systematically investigate the anti-tumor effects of ROF and identify its key molecular targets.

**Materials and methods:**

Anti-tumor activities of ROF were assessed using CCK-8, colony formation, flow cytometry, wound healing, and Transwell assays, respectively. An integrated approach combining network pharmacology, transcriptomic analysis with machine learning was employed to identify primary targets. The Kaplan-Meier survival and ROC curve analyses also evaluated the targets’ clinical outcomes and tumor microenvironment through the Cancer Genome Atlas (TCGA) data and single-cell RNA sequencing. The confirmed experimentally via RT-qPCR, Western blot, and immunofluorescence. The drug-target interaction was characterized by molecular docking and dynamics simulations. Finally, the *in vivo* antitumor efficacy and the safety of ROF were assessed in an H358 xenograft mouse model.

**Results:**

ROF potently inhibited lung cancer cell proliferation (IC_50_: 15.35–33.84 µM), migration, and invasion, while inducing G_2_/M phase arrest and apoptosis (increased Bax/Bcl-2 ratio). ROF also impaired metastatic potential as evidenced by upregulated E-cadherin and downregulated N-cadherin *in vitro*. EPHB2 was identified as the most therapeutically relevant, showing high diagnostic value (AUC=0.856) and a significant correlation with poor patient survival. The experimental validation confirmed that ROF downregulates EPHB2 expression at both the mRNA and protein levels in a dose-dependent manner. Molecular docking and dynamics simulations predicted a stable, high-affinity interaction between ROF and the EPHB2 protein. Importantly, ROF treatment significantly suppressed tumor growth *in vivo* without discernible toxicity.

**Conclusion:**

Rhoifolin exerts potent and selective anti-lung cancer activity by directly targeting and downregulating EPHB2, providing a strong rationale for its further development as a novel therapeutic agent for lung cancer.

## Graphical Abstract

**Figure:**
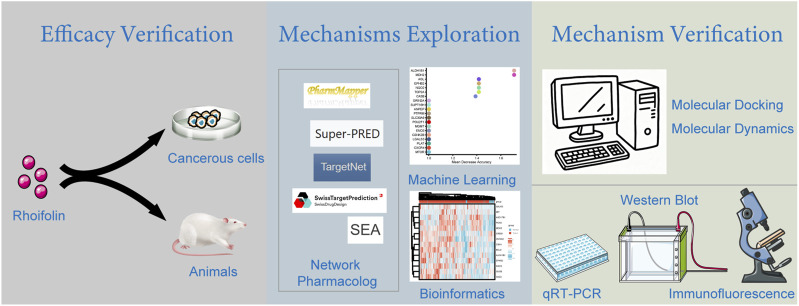

## 1 Introduction

Lung cancer remains the leading cause of cancer-related mortality worldwide and represents one of the most frequently diagnosed malignancies (Brody, 2020). Lung cancer accounted for 2.48 million new cases (12.4% of all cancer diagnoses) and 1.82 million deaths (18.7% of all cancer deaths) according to GLOBOCAN (Bray et al., 2024). The data reflect an increase over 2012, when 1.8 million new cases and 1.6 million deaths were reported (Bade and Dela Cruz, 2020). Lung cancer patients continually exhibit a low 5-year survival rate, despite advancements in various therapeutic approaches. Standard first-line interventions, including surgery, radiotherapy, and chemotherapy, are often limited by issues such as treatment resistance, drug scarcity, and severe adverse effects that may promote tumor recurrence or metastasis (Alsatari et al., 2025; Cangut et al., 2025; Pradhan et al., 2025; Rami-Porta et al., 2016). Therefore, identifying and developing novel inhibitors with enhanced efficacy for lung cancer treatment is urgently needed.

Rhoifolin (ROF) is a flavonoid widely distributed across the plant kingdom, especially within the Rutaceae family and citrus species, and is recognized as a dietary constituent due to its occurrence in edible plants (Alam et al., 2022; Kiliç et al., 2025; Sohi et al., 2025). ROF, chemically identified as apigenin-7-O-β-neohesperidoside, possesses a molecular formula of C27H30O14 and the molecular weight of 578.53 g/mol. It is typically isolated from methanol as a yellow amorphous powder or needle-like crystals (Kiliç et al., 2025). Accumulating evidences indicate that ROF exhibits various bioactivities, particularly potent cytotoxic and anticancer effects, which can induce cancer cell death and inhibiting proliferation, thereby positioning it as a promising anticancer candidate. For instance, ROF could significantly suppresses HepG2 and HuH7 cell proliferation, induces apoptosis, and triggers S-phase cell cycle arrest, while effectively inhibiting tumor growth without apparent toxicity (Chen et al., 2025). Similarly, ROF exerts anti-pancreatic cancer effects through the modulation of the AKT/JNK/caspase-3 and TGF-β2/SMD2 signaling pathways (Zheng et al., 2022). Furthermore, in breast cancer, ROF inhibition of Ezrin phosphorylation and its interaction with PODXL to markedly reduces cell migration, disrupts actin cytoskeleton organization, and suppresses TGF-β1-induced epithelial-mesenchymal transition (EMT) (Xiong et al., 2021). Although the anticancer activities of ROF have been well-documented across various tumor types, its potential role in lung cancer remains unexamined, and its specific molecular targets in this context have not yet been identified.

Therefore, this study was designed to systematically investigate the anti-cancer activity of ROF in lung cancer and identify its key molecular targets. The ultimate goal is to elucidate its underlying mechanism and provide a strong rationale for its development as a novel therapeutic agent in lung cancer.

## 2 Materials and methods

### 2.1 Cell culture and drug treatment

Six human lung cancer cell lines (A549, H358, DMS114, H446, H1299, CALU1), one murine Lewis lung carcinoma (LLC) cell line, and one normal cell line (BEAS2B cell lines) were obtained from the Shanghai Cell Bank of the Chinese Academy of Sciences. We maintained all cell lines at 37 °C in humidified incubators with 5% CO_2_. Culture media were selected based on the biological characteristics of each cell line. CALU1 cells were cultured in MEM medium (Gibco) supplemented with 10% fetal bovine serum (FBS) (MCE, Cat. No.: HY-T1000), 1% non-essential amino acids (NEAA) (MERCK, Cat. No.: M7145), and 1 mM sodium pyruvate (NaP) (MERCK, Cat. No.: S8636); A549 cells were cultured in F12K medium (Gibco) supplemented with 10% FBS and 100 U/mL penicillin-streptomycin (YEASEN, Cat. No.: 60162ES76); H358, DMS114, H446 and H1299 cells were cultured in RPMI-1640 medium (MERCK, Cat. No.: R8758) supplemented with 10% FBS and 100 U/mL penicillin-streptomycin; and LEWIS and BEAS2B cells were cultured in DMEM (Procell, Cat. No.: PM150210) supplemented with 10% FBS and 100 U/mL penicillin-streptomycin. In all tests, cells were inoculated in suitable culture tubes and allowed to adhere overnight. Cells were then treated with different doses of Rhoifolin (MCE, Cat. No.: HY-N0755), which was dissolved in DMSO. The DMSO concentration in the control group was maintained at 0.1%. Following treatment, cells were cultured for the indicated time points before being harvested for next analysis.

### 2.2 Cell viability and IC_50_ determination

The Cell Counting Kit-8 (CCK-8) assay measured cell viability. Cells were seeded at 5,000/well in 100 μL culture media in 96-well plates. After overnight incubation for adhesion, fresh medium with serially diluted ROF was added. The experiment contained a 0.1% DMSO vehicle control group.CCK-8 (Sigma-Aldrich, Cat. No.: 96992) was added to the well, and plates were incubated for another hour at 37 °C. The absorbance was then measured at OD 450 nm. All experiments were performed in triplicate. The half-maximal inhibitory concentration (IC_50_) values were calculated by fitting the dose-response data to a non-linear regression model using GraphPad Prism 9 (GraphPad Software, La Jolla, CA).The formula used was as former reported (Fu et al., 2025).

### 2.3 Colony formation assay and morphological observation

The colony-forming ability of cells was evaluated by seeding 200 cells/well in 6-well plates. Cells were treated with ROF or DMSO for 14 days after overnight attachment. Colonies were fixed with 4% paraformaldehyde (Sigma-Aldrich, Cat. No.: F8775), stained with 0.1% crystal violet, and imaged. ImageJ was used to count colonies and calculate the colony survival fraction as a proportion of the vehicle-treated control group.

Morphological Observation was detected as follows: In 24-well plates, H358 and H1299 cells were planted at a density of 2 × 10^4^ cells/well and cultivated overnight for attachment. An inverted fluorescence microscope was used to observe the cells after 24 h of treatment with ROF or DMSO.

### 2.4 Wound healing assay for cell migration

H358 and H1299 cells were planted in 6-well plates at a density of 5 × 10^5^ cells/well, and a uniform scratch was made in the center of each well using a sterile 200 μL pipette tip (Wu et al., 2025). After washing, cells were cultured in serum-free medium containing either ROF or a vehicle control. After wound closure, images were collected at 0 h, 24 h, and 48 h. The proportion of wound closure compared to scratch breadth measured cell migration into the wound.

### 2.5 Transwell assay for cell invasion

Transwell inserts were first coated with Matrigel (Beyotime, Cat. No.: C0383-5 mL, 50 μL, 1:8 dilution). H358 and H1299 cells, pre-treated for 24 h with ROF or DMSO, were resuspended in serum-free medium (5 × 10^4^ cells/mL) and 200 µL was seeded into the upper chambers. The lower chambers contained complete medium with 10% FBS. After 24 h of incubation, invaded cells were then fixed with 4% paraformaldehyde, stained with 0.1% crystal violet (Justus et al., 2023). Invasion was quantified by counting stained cells in five random fields/well.

### 2.6 Flow cytometric analysis of the cell cycle

A 24-h treatment with ROF or DMSO was followed by the harvesting and fixing of H358 and H1299 cells in 70% ethanol overnight at 4 °C. The fixed cells were then washed and stained with a PI/RNase A solution (Thermo Fisher, Cat. No.: (F10797) for 30 min. Cell cycle profiles were acquired and analyzed using a flow cytometer.

### 2.7 Apoptosis detection by flow cytometry

After 24-h treatment with ROF or DMSO, apoptosis was assessed in H358 and H1299 cells. The cells were harvested and stained with an Annexin V/PI apoptosis detection kit (NEOBIOSCIENCE, Cat. No.: FAK011.100) according to the manufacturer’s instructions.

### 2.8 ROF target prediction

The chemical structure of ROF (PubChem ID: 5282150) was obtained from the PubChem database (https://pubchem.ncbi.nlm.nih.gov/) in SDF format. Potential targets of ROF were predicted using five established online platforms with specific screening criteria:

PharmMapper online analysis platform (https://www.lilab-ecust.cn/pharmmapper/) (Wang et al., 2017): Employing an inverse pharmacophore mapping approach, targets with a Norm Fit Score ≥0.6 were selected. The SuperPred (https://prediction.charite.de/index.php) (Dunkel et al., 2008): Integrating molecular similarity and fragment-based matching, targets with a probability score ≥0.5 and model accuracy ≥ 80% were included. The TargetNet (http://targetnet.scbdd.com/) (Yao et al., 2016)): Based on large-scale QSAR models rather than docking, targets with a predicted probability ≥0.5 were retained. SWISS Target Prediction (Daina et al., 2019; Daina and Zoete, 2024; Gfeller et al., 2013): Combining 2D and 3D similarity measures with known ligands, the targets with probability > 0.1 were selected. SEA (Similarity Ensemble Approach, http://sea.bkslab.org/) (Keiser et al., 2007): Comparing the chemical structure of ROF against target ligand sets, targets with P ≤ 0.05 were considered significant. For all predictions, the species was set to “*Homo sapiens*” to match the subsequent lung cancer cell studies. After predictions, the potential target lists were consolidated and deduplicated using Microsoft Excel. The candidate targets were then imported into Cytoscape 3.9.1 to construct a “Compound-Target” network visualizing the interactions between ROF and its targets. Gene ontology (GO) and Kyoto Encyclopedia of Genes and Genomes (KEGG) pathway enrichment analyses were conducted with the targets, setting P < 0.05 and FDR <0.05 as significant.

### 2.9 Machine learning to identify hub genes of ROF

Differentially expressed genes from the lung cancer-related transcriptomic dataset GSE226774 (obtained from the GEO database, which includes lung cancer and normal lung tissue samples) were selected based on the criteria of |log_2_FC| > 1 and Adjusted P < 0.05. The intersection of these genes with the ROF candidate targets identified previously was used to construct the core features for model development. A Random Forest algorithm was employed in R 4.3.1 (using the caret package) to build the predictive model. Key model parameters were optimized through 10-fold cross-validation, including the number of decision trees (ntree, range 100–1000) and the maximum depth of the decision trees (maxdepth, range 5–30). The optimal parameter combination was determined based on the highest predictive accuracy. Model performance was evaluated using accuracy, sensitivity, specificity, and the area under the ROC curve (AUC). A Mean Decrease Gini importance score greater than 1.4 was considered indicative of the critical potential core targets of ROF.

### 2.10 Bioinformatics analysis of hub genes

Differential expression analysis, Kaplan-Meier curve analysis, and ROC curve analysis were performed using XianTao Academic (https://www.xiantaozi.com/). Single-cell analysis was carried out using the Single-cell and Spatially-resolved Cancer Resources (Deng et al., 2024), analyzing the expression distribution of core targets in various cell subpopulations (e.g., tumor cells, immune cells, stromal cells).

### 2.11 EPHB2 mRNA expression analysis

Total RNA was extracted from ROF-or DMSO-treated H358 cells and H446 cells using TRIzol (Invitrogen, Cat. No.: 15596026). and cDNA was synthesized using the reverse transcription kit (GeneCopeia, Cat. No.: QP056T), with total RNA as the template. The expression of EPHB2 was then quantified by qRT-PCR using a SYBR Green master mix (Servicebio, Cat. No.: G3320-05).

The EPHB2 primers were designed as follows:

upstream 5′- CCA​CAG​CCA​TAA​AAA​GCC​CC-3′ and downstream 5′- CTT​CAT​GCC​TGG​GGT​CAC​TT-3′, with β-actin primers used as the reference gene (sequences: upstream 5′- AGC​ATC​CCC​CAA​AGT​TCA​CAA -3′ and downstream 5′-TGG​GGT​GGC​TTT​TAG​GAT​GG -3′; primers synthesized by Qingke Biotechnology Co., Ltd.).

40 cycles of 95 °C for 30 s (pre-denaturation), 95 °C for 5 s (denaturation), and 60 °C for 30 s (annealing) were followed by a final 95 °C for 15 s, 60 °C for 1 min, and 95 °C for 15 s to construct a melting curve to check primer specificity. Three technical and three biological replicates were done (Wang et al., 2021).

### 2.12 Immunofluorescence to detect EPHB2 protein localization and expression

ROF or DMSO for 24 h was used to treat H358 cells on coverslips in 24-well plates (1 × 10^4^ cells/well). Cells were fixed with 4% paraformaldehyde, permeabilized with 0.5% Triton X-100, and blocked with 5% BSA. After washing three times with PBS, cells were incubated overnight at 4 °C with a primary antibody against EPHB2 (1:200; SCBT, Cat.: 2D12C6), followed by 1 hour incubation with a fluorescent secondary antibody (1:500; Abcam, Cat. No.: ab279292). The nuclei were stained with DAPI. Images were captured using a Zeiss LSM 880 confocal microscope. Three random fields were captured for analysis.

### 2.13 E-cadherin,N-cadherin, Bax, Bcl-2, CDK1, cyclin B1 and EPHB2 protein expression analysis

Total protein was separated by SDS-PAGE and transferred to PVDF membranes. After blocking membranes, primary antibodies were incubated overnight at 4 °C against (ECAD (1:1000; abcam, Cat. No.: ab40772)NCAD (1:1000; abcam, Cat. No.: ab76011) BAX (1:1000; MCE, Cat. No.: YA591)BCL2 (1:1000; MCE, Cat. No.: YA592)) EPHB2 (1:1000; SCBT, Cat. No.: 2D12C6). CDK1 (1:1000; HUABIO,Cat.No.:ET1607-51), Cyclin B1 (1:1000; HUABIO, Cat.No.:ET1608-27). An HRP-conjugated secondary antibody (1:10,000) was incubated for 1 h. Protein bands were visualized and imaged.

### 2.14 Molecular docking to validate binding of ROF and EPHB2

The 3D structures of ROF (PubChem ID: 5282150) and the EPHB2 protein (PDB ID: 3ZFM) were prepared using AutoDock Tools v1.5.6. This preparation involved adding polar hydrogens and Gasteiger charges to both molecules and removing non-essential components (e.g., water, original ligands) from the protein. The prepared structures were saved in PDBQT format. Docking was then performed with AutoDock Vina, with the search grid (20 × 20 × 20 Å) centered on the known active site of EPHB2. The resulting binding poses were ranked by their affinity scores, with a binding energy ≤ −5.0 kcal/mol considered significant. The lowest energy conformation was selected for detailed analysis of its interactions with the protein’s active site residues, which was visualized using PyMOL v2.5.

### 2.15 Molecular dynamics (MD) simulation to validate complex stability

Molecular dynamics simulations of the EPHB2-ROF complex were conducted using Gromacs 2023.2 software with the AMBER99SB-ILDN force field to study the dynamic response of the protein-ligand complex. The complex was placed in a cubic box with a 1.0 nm distance between the edge of the box and the molecule. Periodic boundary conditions were applied, and the system was neutralized by replacing some water molecules with Na+ and Cl− ions at physiological concentration (0.15 M). Energy minimization was performed using the steepest descent method to reduce the system’s energy to less than 1000 kJ/(mol·nm). The system was pre-equilibrated in both NVT and NPT ensembles before undergoing free molecular dynamics simulation. The simulation ran for 5,000,000 steps with a 2-fs time step, for a total of 100 ns. After the simulation, the trajectories were analyzed using built-in Gromacs tools to calculate root mean square deviation (RMSD), root mean square fluctuation (RMSF), and radius of gyration, as well as free energy (MM/PBSA) and free energy distribution.

### 2.16 *In vivo* xenograft tumor model and drug efficacy evaluation

BALB/c nude mice were purchased from Beijing Vital River Laboratory Animal Technology Co., Ltd. (females, 4–6 weeks old, 18–22 g). A week after acclimatization, mice were divided into four groups (finally, four mice per group): control (DMSO), low-dose ROF (10 mg/kg), medium-dose ROF (20 mg/kg), and high-dose ROF (40 mg/kg. A single-cell suspension of H358 cells (5 × 10^6^ cells in 100 μL of PBS) into the subcutaneous space of the right armpit of BALB/c nude mice to establish a xenograft model. After 5 days, ROF was dissolved in DMSO and administered intraperitoneally (i.p.) at doses of 10, 20, and 40 mg/kg. The administration was performed once daily for a period of 14 days. Tumor volume and body weight were measured every 2 days. Tumor growth was monitored by measuring tumor length (a) and width (b), with tumor volume calculated using the formula: V = 0.5 × a × b^2^. Tumor growth curves and body weight change curves were plotted. Values are expressed as Mean ± SD. After euthanizing mice, tumors and main organs (heart, liver, spleen, lungs, kidneys) were removed, weighed, and photographed. Histological examination was performed on tissues fixed in 4% paraformaldehyde.

Paraffin-embedded tissue sections (5 μm) were prepared, mounted on slides, and baked. The sections were then deparaffinized with xylene and rehydrated through graded ethanol. The HE staining protocol including staining with hematoxylin (5 min), differentiation in 1% acidic ethanol (30 s), bluing in water (5 min), and counterstaining with eosin (2 min). Staining was followed by dehydration in graded ethanol, clearing in xylene, and mounting in neutral resin. Histopathological examination was conducted using a light microscope, with five random fields per group photographed for analysis.

### 2.17 Statistical analysis

All experimental data were analyzed with GraphPad Prism 9.0 software. Data are presented as mean +standard deviation (‾x ± s). Two groups were compared using the independent sample t-test, and multiple groups were compared using the one-way ANOVA. Statistical significance was defined as a p-value of less than 0.05.

## 3 Results

### 3.1 ROF inhibits proliferation of lung cancer cells *in vitro*

**FIGURE 1:**
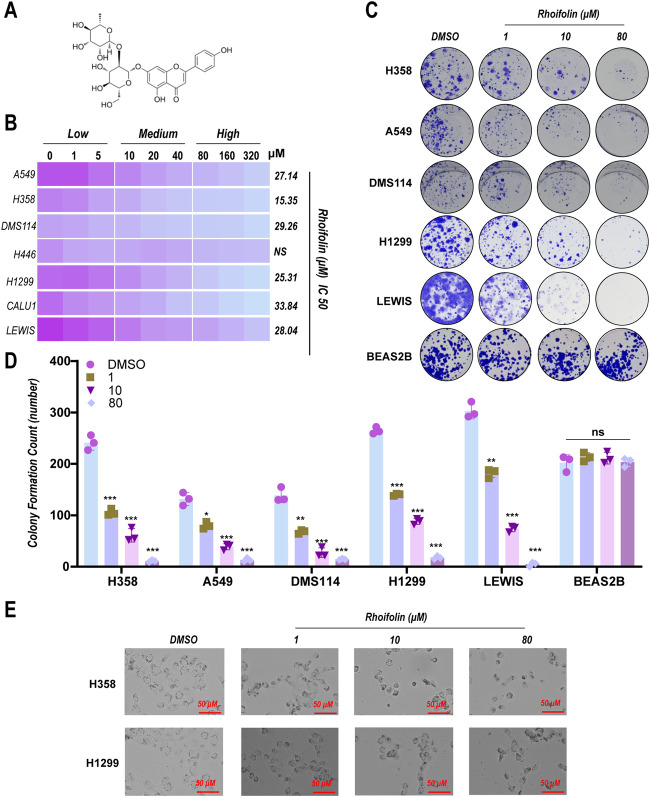
Inhibitory effect of Rhoifolin on lung cancer cell proliferation. **(A)** Chemical structure of Rhoifolin. **(B)** CCK-8 assay showing the effect of Rhoifolin on the proliferation of seven lung cancer cell lines and corresponding IC_50_ values. **(C,D)** Colony formation assays showing the effect of Rhoifolin on the colony formation ability of A549, H358, DMS114, H1299, LEWIS lung cancer cells and BEAS2B normal bronchial epithelial cells. **(E)** Morphological changes of H358 and H1299 cells observed under an inverted microscope after Rhoifolin treatment. Statistical significance was determined using p-values, with *p < 0.05, **p < 0.01, and ***p < 0.001 indicating significant differences between groups. “ns” denotes no statistical significance.

We first investigated the anti-proliferative activity of ROF (Figure 1A) on a panel of seven lung cancer cell lines using the CCK-8 assay. ROF demonstrated significant, dose-dependent inhibitory effects on six out of 7 cell lines, with IC_50_ values between 15.35 µM and 33.84 µM. The H358 cell line exhibited the highest sensitivity to ROF, with an IC_50_ value of 15.35 µM, whereas the H446 cell line demonstrated significant resistance to the compound’s effects (Figure 1B).

To validate these findings we then performed a colony formation assay using A549, H358, DMS114, H1299, LEWIS lung cancer cells, and normal human bronchial epithelial BEAS2B cells. In line with the CCK-8 results, ROF significantly reduced colony formation of all tested cell lines. Crucially, ROF displayed preferential cytotoxicity towards cancer cells, as concentrations that were highly effective against lung cancer lines showed no significant impact on the viability of non-malignant human bronchial epithelial BEAS2B cells (Figures 1C,D). Additionally, morphological changes in H358 and H1299 cells treated with ROF were observed using an inverted microscope. Compared to the control group, ROF-treated cells exhibited significant shrinkage, rounding, and detachment (Figure 1E), further confirming its inhibitory effect on cell proliferation.

### 3.2 ROF inhibits migration and invasion of lung cancer cells *in Vitro*

**FIGURE 2:**
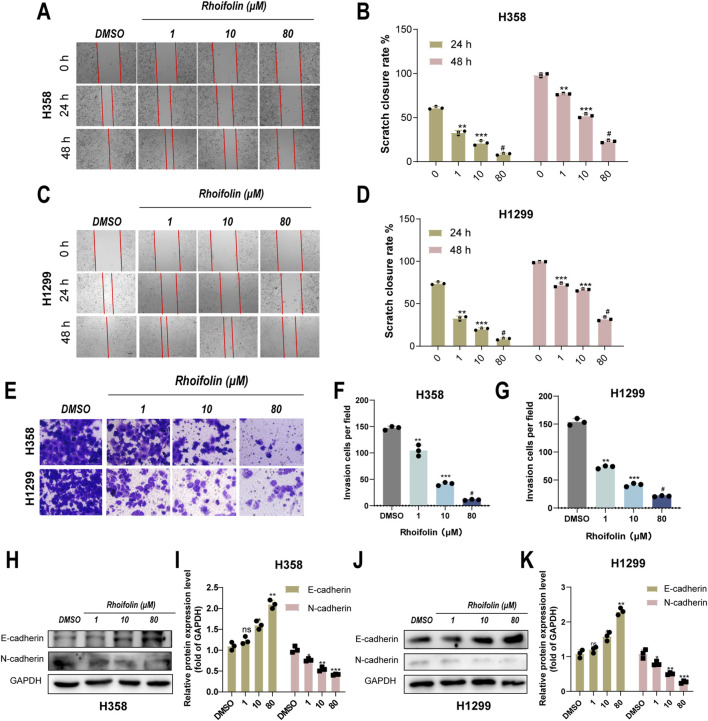
Effect of Rhoifolin on lung cancer cell migration and invasion. **(A–D)** Scratch assay showing the effect of Rhoifolin on the migration of H358 and H1299 cells. **(E–G)** Transwell invasion assays showing the effect of Rhoifolin on the invasion ability of H358 and H1299 cells. **(H–K)** Western blot analysis of EMT-related markers E-cadherin and N-cadherin in H358 and H1299 cells treated with Rhoifolin. Statistical significance was determined using p-values, with *p < 0.05, **p < 0.01, and ***p < 0.001 indicating significant differences between groups. “ns” denotes no statistical significance.

The effect of ROF on the migration and invasion of lung cancer cells was evaluated using distinct assays. The effect of ROF on the migration of lung cancer was evaluated by performing a scratch assay with H358 and H1299 cells. Compared to the control group, the ROF treatment groups (1, 10, 80 μM) exhibited significantly lower wound healing rates at 24 and 48 h, with a concentration-dependent decrease in healing rate (Figures 2A–D). Additionally, the invasive potential was examined using Transwell invasion assays, which further confirmed that the number of invasive cells was markedly decreased after ROF treatment (Figures 2E–G). To explore the molecular mechanism underlying ROF’s effects on migration and invasion, protein expression levels of EMT markers, E-cadherin (epithelial marker) and N-cadherin (mesenchymal marker), were assessed via Western blotting. The results revealed that ROF treatment significantly elevated E-cadherin expression and decreased N-cadherin expression in both H358 and H1299 cells (Figures 2H–K).

### 3.3 ROF induces G2/M cell cycle arrest and apoptosis of lung cancer cells *in Vitro*

**FIGURE 3:**
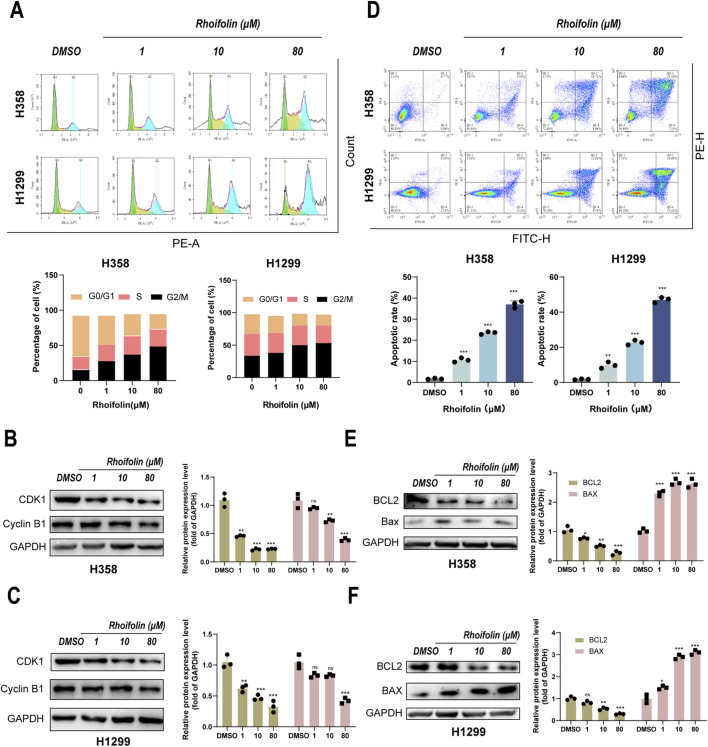
Effect of Rhoifolin on lung cancer cell cycle and apoptosis. **(A)** Flow cytometry analysis showing the effect of Rhoifolin on the cell cycle distribution of H358 and H1299 cells. **(B,C)** Western blot analysis of cell cycle proteins (CDK1,Cyclin B1) in the Rhoifolin-treated H358 and H1299 cells. **(D)** Flow cytometry analysis of apoptosis in H358 and H1299 cells treated with Rhoifolin. **(E,F)** Western blot analysis of apoptotic regulatory proteins (Bax and Bcl-2) in Rhoifolin-treated H358 and H1299 cells. Statistical significance was determined using p-values, with *p < 0.05, **p < 0.01, and ***p < 0.001 indicating significant differences between groups. “ns” denotes no statistical significance.

Considering that ROF inhibits cell proliferation, we next used flow cytometry to assess its impact on cell cycle progression and apoptosis. Cell cycle analysis demonstrated that ROF treatment (1, 10, 80 µM) resulted in a significant, dose-dependent accumulation of H358 and H1299 cells in the G_2_/M phase. The observed depletion of the G_0_/G_1_ phase population indicates that ROF effectively induces G_2_/M phase cell cycle arrest, consequently inhibiting cell proliferation (Figure 3A). We further examined the expression of key G2/M regulatory proteins by Western blotting. As shown in Figures 3B,C, the protein levels of CDK1 and Cyclin B1 were significantly decreased in both cell lines. The suppression of these cyclin-CDK complexes, which are essential for mitotic entry, aligns with the accumulation of cells in the G2/M phase observed in our cell cycle analysis. As shown in Figure 3D, ROF treatment markedly elevated the total apoptosis rate in both H358 and H1299 cells. The protein levels of the pro-apoptotic Bax were increased, whereas the levels of the anti-apoptotic Bcl-2 were decreased, as assessed by Western blotting (Figures 3E,F).

### 3.4 Network pharmacology and machine learning to identify potential critical genes of ROF

**FIGURE 4:**
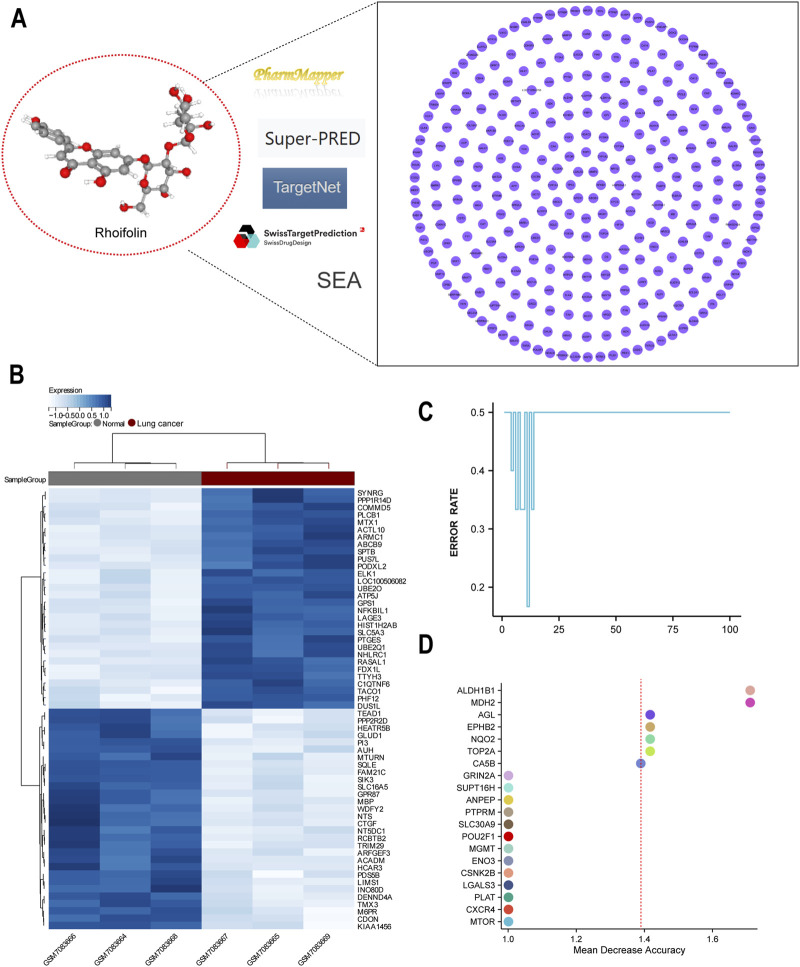
Network pharmacology and machine learning to identify core targets of Rhoifolin. **(A)** Prediction of Rhoifolin’s potential targets from online databases. **(B)** Heatmap analysis based on the lung cancer-related transcriptomic dataset GSE226774. **(C,D)** Random Forest algorithm-based prediction model to assess target gene importance based on Mean Decrease Accuracy.

We utilized a network pharmacology approach to systematically identify the molecular targets associated with the anti-tumor actions of ROF. By integrating predictions from five independent online public databases, a total of 320 potential targets were identified after deduplication (Figure 4A). GO enrichment analysis revealed that targets were predominantly enriched in cellular components such as “extracellular exosome,” “cytosol,” “extracellular space,” and “membrane-enclosed lumen.” KEGG pathway enrichment analysis indicated that targets were significantly enriched in metabolic pathways, cancer-related pathways (e.g., “Pathways in cancer,” “PD-L1 expression and PD-1 checkpoint pathway in cancer”), and signal transduction pathways (e.g., “MicroRNAs in cancer,” “Complement and coagulation cascades”) (Supplementary Figure S1). To identify this critical targets, machine learning analysis was performed using lung cancer transcriptomic data (GSE226774). The heatmap analysis demonstrated distinct clustering between lung cancer and normal lung tissue samples, suggesting high data reliability (Figure 4B). Random Forest modeling was used to evaluate the importance of target genes based on their expression levels, with Mean Decrease Accuracy (MDA) values above 1.4 for six genes: ALDH1B1, MDH2, AGL, EPHB2, NQO2, and TOP2A (Figures 4C,D). These six genes were identified as potential core targets of ROF’s anti-lung cancer action.

### 3.5 Bioinformatics analysis of core genes

**FIGURE 5:**
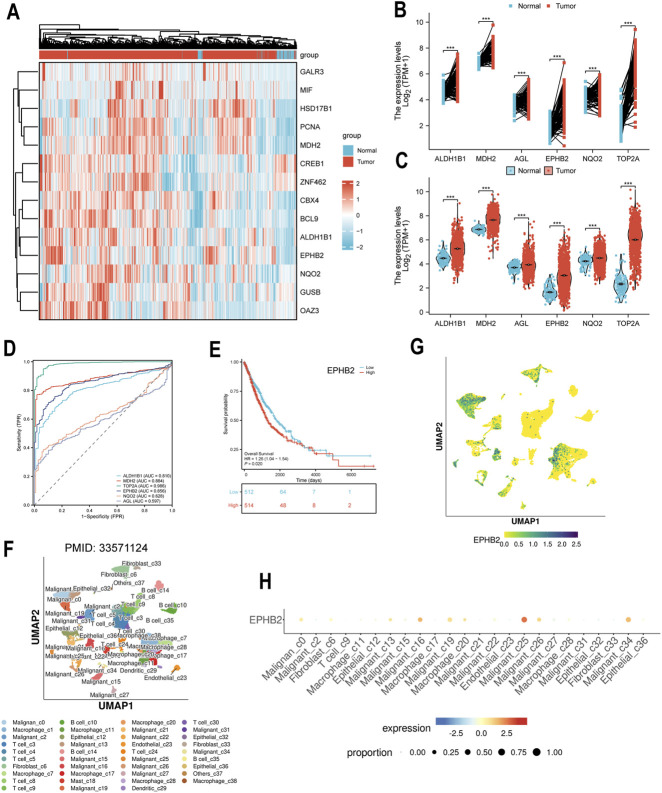
Bioinformatics analysis of potential core genes. **(A)** mRNA expression levels of potential core genes (ALDH1B1, MDH2, AGL, EPHB2, NQO2, TOP2A) in the TCGA lung cancer database. **(B,C)** Paired and unpaired sample analysis of core gene expression. **(D)** ROC curve analysis to evaluate the diagnostic value of core genes in lung cancer. **(E)** Kaplan-Meier survival analysis of core genes. **(F,G)** UMAP-based dimensionality reduction visualization of EPHB2 expression in the lung cancer single-cell transcriptome dataset. **(H)** Quantitative analysis of EPHB2 expression in different cell subpopulations (malignant, T cells, macrophages, epithelial cells).

To verify the clinical significance of the core genes (ALDH1B1, MDH2, AGL, EPHB2, NQO2, TOP2A) in lung cancer, their expression levels in the TCGA lung cancer database were analyzed. The mRNA transcription levels of all six genes were considerably greater in lung cancer tissues than normal lung tissues (Figure 5A), with consistent findings from paired and non-paired sample analyses (Figures 5B,C). ROC curve analysis showed that TOP2A had the highest AUC (0.986), followed by MDH2 (0.884), EPHB2 (0.856), ALDH1B1 (0.810), while NQO2 (0.628) and AGL (0.597) had AUC values below 0.7 but still higher than random chance (AUC=0.5) (Figure 5D). Kaplan-Meier survival analysis revealed that only EPHB2 expression levels were substantially correlated with overall survival (OS), with individuals showing high EPHB2 expression demonstrating markedly shorter OS compared to those with low expression (Log-rank P < 0.05) (Figure 5E). No significant association between the other five genes and patient prognosis was observed (Supplementary Figure S2).

To further investigate the cellular origin of EPHB2 expression within the tumor microenvironment, we conducted an analysis of single-cell RNA sequencing dataset from lung cancer patients. UMAP visualization demonstrated that EPHB2 expression was predominantly localized to malignant cell clusters, with limited expression observed in adjacent immune and stromal cell populations (Figures 5F,G). Quantitative analysis further confirmed that EPHB2 expression was significantly increased in tumor cell subpopulations (Malignant_c16, Malignant_c25, Malignant_c34) compared to immune and stromal cell subpopulations lung cancer (Figure 5H), providing a theoretical basis for further validation of ROF targeting EPHB2 in lung cancer.

### 3.6 ROF targets EPHB2, reduces EPHB2 mRNA expression and protein abundance

**FIGURE 6:**
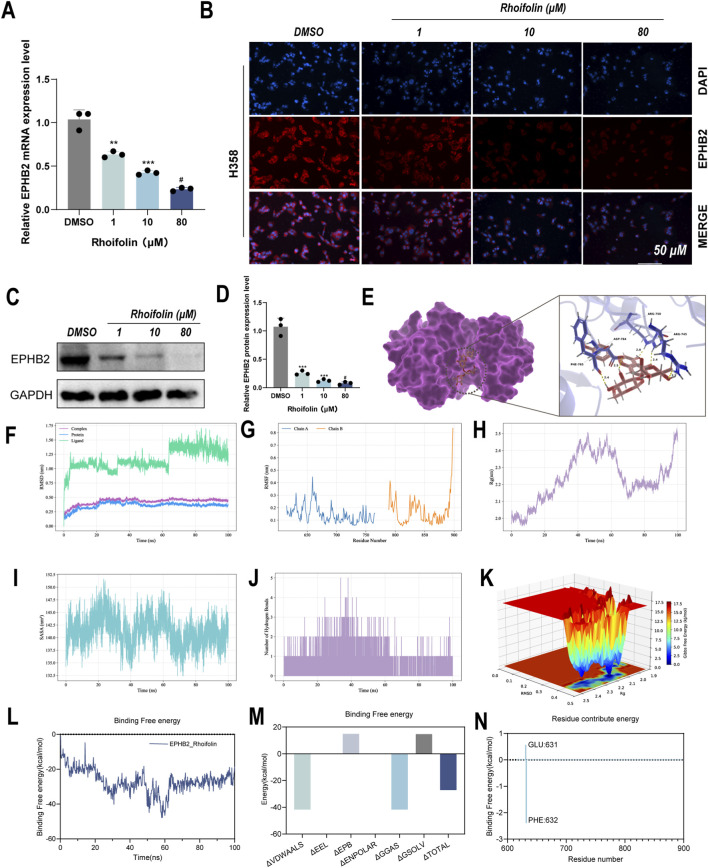
Verification of Rhoifolin potential targeting EPHB2. **(A)** qRT-PCR analysis of EPHB2 mRNA expression in H358 cells treated with Rhoifolin. **(B)** Immunofluorescence observation of EPHB2 protein localization and expression. **(C,D)** Western blot analysis of EPHB2 protein expression in H358 cells treated with Rhoifolin. **(E)** Molecular docking simulation of Rhoifolin binding to EPHB2. **(F)** RMSD analysis of the protein-ligand complex during the simulation. **(G)** RMSF analysis of the protein-ligand complex during the simulation. **(H)** Radius of gyration (Rg) analysis of the protein-ligand complex. **(I)** SASA analysis of the protein-ligand complex. **(J)** Hydrogen bond analysis of the protein-ligand complex during the simulation. **(K)** Free energy landscape of the protein-ligand complex during the simulation. **(L)** Dynamic change in binding free energy of the Rhoifolin-EPHB2 complex during 100 ns simulation. **(M)** Total binding free energy of the Rhoifolin-EPHB2 complex. **(N)** Free energy decomposition analysis of key residues in the Rhoifolin-EPHB2 complex.

Based on the above findings, we hypothesized that EPHB2 is a primary molecular target through which ROF exerts its anti-tumor effects. qRT-PCR analysis revealed that ROF treatment resulted in a significant, dose-dependent reduction in EPHB2 mRNA levels relative to the vehicle control in the sensitive H358 cells (Figure 6A). In H446 cells, which exhibit significant resistance to ROF, EPHB2 mRNA levels showed no statistically significant alteration relative to the control (Supplementary Figure S3). This observation, coupled with the downregulation seen in sensitive cells, further suggests that EPHB2 serves as a potential primary functional target for the anti-tumor efficacy of ROF. The protein level indicated this transcriptional downregulation. Immunofluorescence staining revealed a significant decrease in EPHB2 protein expression, with the fluorescence signal nearly undetectable at the highest ROF concentration (80 µM) (Figure 6B). The quantitative validation of this finding was achieved through Western blot analysis, demonstrating a clear dose-dependent reduction in EPHB2 protein levels in ROF-treated cells (Figures 6C,D).

### 3.7 Molecular docking and molecular dynamic stimulation analysis of the EPHB2-ROF complex binding

Moreover, the molecular docking simulations were showed that the binding energy between ROF and EPHB2 was −6.43 kcal/mol (≤−5.0 kcal/mol), with ROF forming hydrogen bonds with several amino acid residues, including ASP-764, PHE-765, ARG-745, and ARG-750, with bond lengths ranging from 2.0 to 2.5 Å (Figure 6E). These results suggest a potential binding interaction between ROF and EPHB2.

We further conducted molecular dynamics (MD) simulations of the EPHB2-ROF complex over a 100 ns time period to explore the binding kinetics and stability of the complex (Figure 6E). The EPHB2-ROF complex achieved rapid conformational stability, as evidenced by a stable RMSD of approximately 0.3 nm after 20 ns (Figure 6F), indicating the formation of a highly stable structure. Analysis of Rg and SASA further confirmed that the complex remained highly compact throughout the simulation (Figures 6H,I). The average Rg during the 100 ns simulation was 2.2371 nm (Figure 6H), indicating that the protein remained compact and did not undergo significant relaxation or expansion upon ligand binding. RMSF analysis was performed to evaluate the structural flexibility of the complex. The results showed that ROF’s binding interaction did not significantly disrupt the protein’s key functional sites (Figure 6G), and a persistent network of hydrogen bonds was maintained, signifying a stable binding mode (Figure 6J). Finally, the binding free energy, calculated via the MM/PBSA method, was highly favorable at −27.05 kcal/mol, indicating a strong and spontaneous interaction (Figures 6K–N). Collectively, these simulations demonstrate that ROF forms a durable and energetically stable complex with the EPHB2 protein.

In summary, the molecular dynamics simulation results demonstrate that ROF forms a highly stable complex with EPHB2. Throughout the simulation, ROF remains firmly bound to the active site of EPHB2 without significant dissociation or conformational disruption, showcasing remarkable binding stability and specificity. This stable interaction provides a solid molecular basis for ROF as a potential regulatory agent targeting EPHB2.

### 3.8 *In Vivo* efficacy and preliminary safety evaluation of ROF

**FIGURE 7:**
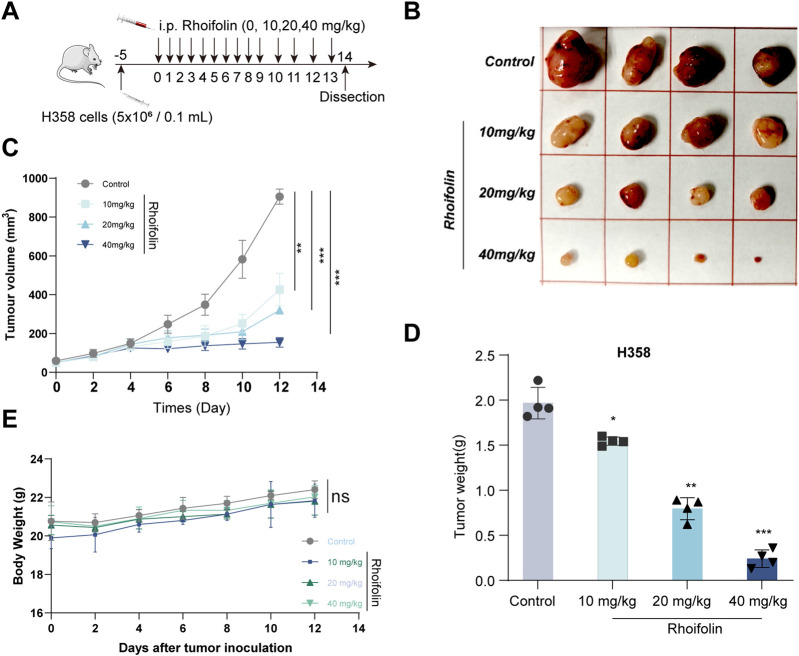
*In vivo* anti-lung cancer efficacy of Rhoifolin. **(A)** Representative schematic of the H358 cell xenograft mouse model and drug treatment groups. A week after acclimatization, mice were divided into four groups (four mice per group): control (DMSO), low-dose ROF (10 mg/kg), medium-dose ROF (20 mg/kg), and high-dose ROF (40 mg/kg). H358 cells (5 × 10^6^ cells in 100 μL of PBS) were inoculated into the subcutaneous space of the right armpit of BALA/c nude mice to establish a xenograft tumor model. After 5 days, ROF was dissolved in DMSO and administered intraperitoneally (i.p.) at doses of 10, 20, and 40 mg/kg. The administration was performed once daily for a period of 14 days. The arrow indicates the start of treatment. Tumor volume and body weight were measured every 2 days. Values are expressed as Mean ± SD **(B)** Representative images of tumors at the endpoint, showing morphological differences among the treatment groups. **(C)** Tumor volume changes at different time points during the experiment. Tumor volumes in the different groups increased in the following order: the ROF high-dose group, medium-dose group, low-dose group, and DMSO control groups. **(D)** Statistical analysis of tumor weights at the endpoint. **(E)** Dynamic monitoring of mouse body weight during the treatment. Statistical significance was determined using p-values, with *p < 0.05, **p < 0.01, and ***p < 0.001 indicating significant differences between groups. “ns” denotes no statistical significance.

**FIGURE 8:**
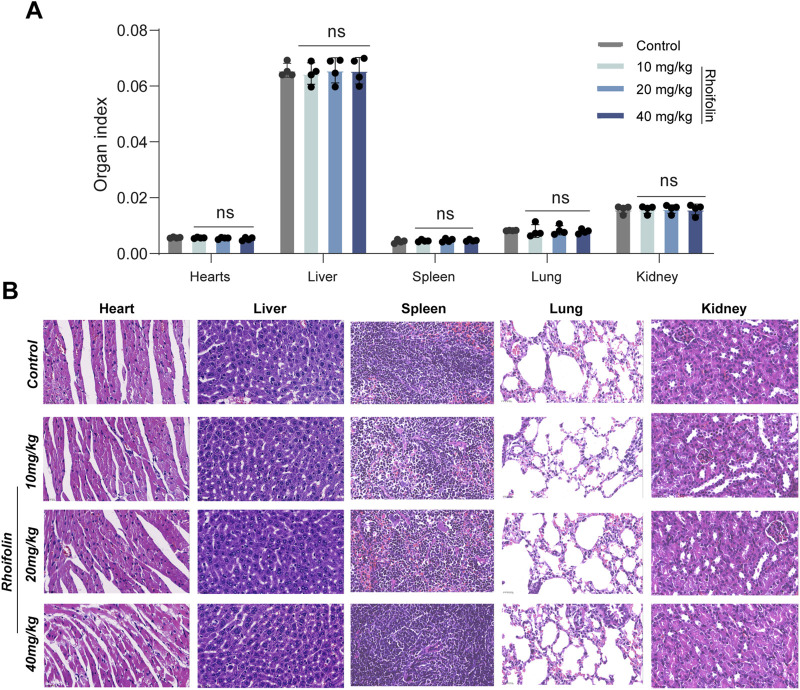
*In vivo* safety evaluation of Rhoifolin. **(A)** Organ index measurements in different treatment groups. **(B)** HE staining showing the pathological morphology of the heart, liver, spleen, lung, and kidney tissues in all groups. Statistical significance was determined using p-values, with *p < 0.05, **p < 0.01, and ***p < 0.001 indicating significant differences between groups. “ns” denotes no statistical significance.

A H358 cell-derived xenograft mouse model was established to evaluate the *in vivo* anti-lung cancer activity of ROF. The tumor volumes in the control group were 904.748 ± 38.916 mm^3^ (Mean ± SD, n = 4), while those in the Rhoifolin-treated groups (10, 20, 40 mg/kg) were 425.813 ± 84.014 mm^3^, 319.953 ± 16.851 mm^3^, and 154.550 ± 24.679 mm^3^, respectively, after 14 days of treatment (p < 0.05). The results demonstrated that, compared to the control group, ROF-treated groups showed significantly reduction in tumor volume and weight across all dosages (Figures 7A–D), thereby confirming the efficacy of ROF in inhibiting the growth of lung cancer xenografts *in vivo*. To evaluate its *in vivo* safety, mouse body weight was monitored throughout the treatment. The data indicated a progressive increase in body weight across all groups (Figure 7E). Furthermore, organ index were measured, and no significant differences were observed between the treatment and control groups (P > 0.05) (Figure 8A). Histological analysis using HE staining revealed no significant pathological damage in the heart, liver, spleen, lung, or kidney tissues of both the treated and control groups, with intact tissue structures (Figure 8B).

## 4 Discussion

Pharmacological treatment remains a crucial approach for lung cancer therapy, however, the efficacy of existing agents is frequently limited by considerable side effects and tumor heterogeneity. This necessitates the discovery of novel therapeutic molecules with improved efficacy and safety profiles. ROF, a natural flavonoid, has shown antitumor potential in several cancers (liver, pancreatic, and breast), however, its role in lung cancer has not been extensively studied. This study, for the first time, systematically elucidates the potent anti-lung cancer activity of ROF and unveils a key molecular mechanism centered on the direct targeting of the EPHB2 receptor tyrosine kinase.

Uncontrolled proliferation, migration, and invasion are interconnected hallmarks that collectively drive malignant progression and metastasis in tumor cells (Hanahan, 2022; Hanahan and Weinberg, 2011). Consequently, focusing on these biological characteristics is essential in anticancer therapy. We found that ROF exhibited notable antitumor effects in lung cancer by inhibiting cell proliferation, migration, and invasion, while simultaneously inducing apoptosis. Firstly, ROF suppressed proliferation in a dose-dependent manner with selectivity cytotoxicity, preserving non-malignant bronchial epithelial cells. Our results further demonstrated that the anti-proliferative effect was driven by a pronounced G_2_/M phase cell cycle arrest and the induction of intrinsic apoptosis, mediated through a shift in the Bax/Bcl-2 ratio and the downregulation of CDK1 and Cyclin B1, key regulators of the G_2_/M transition. As the ROF induced cell cycle arrest at the G_2_/M phase that is particularly significant for cell division and DNA replication, and its blockade can effectively restrict tumor cell proliferation (Smith et al., 2020). Additionally, ROF impaired metastatic potential by reducing cell migration and invasion, a phenotype consistent with the reversal of EMT, as evidenced by upregulated E-cadherin and downregulated N-cadherin. This multi-mechanistic profile positions ROF as a promising multi-faceted therapeutic candidate. These effects were consistent with the known antitumor activities of other flavonoids, which have been documented to suppress cancer progression through mechanisms including cell cycle arrest, apoptosis induction, and modulation of critical signaling pathways (Forni et al., 2021; Hazafa et al., 2020; Kopustinskiene et al., 2020).

To elucidate the molecular mechanism of these effects, we employed an integrated computational and bioinformatic strategy. By integrating network pharmacology with machine learning analysis of clinical transcriptional data. The Random Forest model successfully prioritized six primary targets (ALDH1B1, MDH2, AGL, EPHB2, NQO2, and TOP2A). Further validation utilizing the TCGA database and single-cell RNA-seq data narrowed these candidates down to EPHB2, which was not only highly expressed in tumor tissues but was also significantly associated with patient prognosis and specifically enriched in malignant cell populations. The ROC curve shown the TOP2A, MDH2, EPHB2, and ALDH1B1 possess substantial diagnostic potential, with TOP2A achieving an AUC of 0.986. Kaplan-Meier survival analysis suggested that EPHB2 may function as a negative prognostic biomarker in lung cancer. Single-cell transcriptomic profiling confirmed that EPHB2 expression is predominantly localized to tumor cell subpopulations, highlighting its significance as a critical target. These findings align with existing literature reporting that flavonoids can inhibit EMT and tumor metastasis by modulating tyrosine kinase receptor signaling, consistent with our observation that ROF suppresses lung cancer cell migration and invasion through EMT regulation (Mohammad et al., 2025; Proença et al., 2023). EPHB2, a member of the Eph receptor tyrosine kinase family, has been implicated in promoting tumor progression in lung cancer and other solid malignancies by regulating cell proliferation, migration, invasion, and EMT, with elevated expression correlating with poor patient prognosis (Ebrahim et al., 2021; Liu et al., 2022).

Subsequent experimental and biophysical analyses provided robust support for EPHB2 engagement. ROF treatment downregulated EPHB2 expression at both the transcriptional and translational levels. Molecular docking predicted a favorable binding mode within the EPHB2 active site, which was further validated by extensive molecular dynamics simulations. The ROF-EPHB2 complex exhibited remarkable stability and energetically favorable binding dynamics characterized by persistent hydrogen bonding and a highly negative binding free energy (binding free energy of −27.05 kcal/mol), predominantly driven by electrostatic and van der Waals forces. Per-residue free energy decomposition revealed that PHE632 plays a crucial role in complex formation. The simulations establish a solid biophysical basis for ROF as a direct EPHB2-binding agent, indicating that ROF not only downregulates EPHB2 but also stabilizes the protein, thereby confirming its potential as an anti-lung cancer agent across molecular and cellular levels.

*In vivo* pharmacological evaluation serves as an essential connection between *in vitro* results and prospective clinical application (Kimura, 2005). Crucially, we translated these findings into a physiological context using a H358 xenograft model. ROF administration significantly suppressed tumor growth. This potent anti-tumor activity was achieved without any discernible systemic toxicity, as evidenced by stable body weights, normal organ indices, and the absence of histopathological damage in major organs. Compared with conventional chemotherapeutic agents, the natural small molecule ROF demonstrates potent tumor-suppressive activity while exhibiting low systemic toxicity, highlighting the unique advantages of natural product-based therapeutics. Furthermore, this work establishes an initial “mechanism–efficacy–safety” framework for ROF, providing a solid foundation for subsequent investigations. The “high-efficacy, low-toxicity” profile underscores the unique advantages of natural product-based therapeutics. However, it should be noted that this study represents a preliminary exploration. While pharmacological and computational evidence supports EPHB2 as a target, further genetic validation (knockdown or overexpression) remains to be conducted in the future studies. Furthermore, our current *in vivo* findings primarily focused on tumor growth inhibition and systemic safety. Building on this foundation, several important future directions are warranted: specifically investigating the potential of ROF in preventing lung cancer metastasis, optimizing ROF derivatives for enhanced EPHB2 affinity and exploring synergistic combinations with existing therapies, such as immune checkpoint inhibitors.

Taken together, this study systematically elucidates a novel anti-lung cancer mechanism for ROF, demonstrating that it directly targets and downregulates the clinically relevant EPHB2 receptor. This interaction interferes with essential oncogenic processes, including proliferation and apoptosis, ultimately leading to potent and safe tumor suppression *in vivo*. Our work not only provides a solid preclinical rationale for the development of ROF as a novel therapeutic agent for lung cancer but also highlights EPHB2 as a promising druggable target.

## Data Availability

The GEO public datasets used in this study are available in the data repository under the accession number GSE226774, which contains the microarray data. Further inquiries can be directed to the corresponding author(s).

## Supplementary material

SUPPLEMENTARY FIGURE S1 GO cell component (CC) and KEGG pathway enrichment analysis of Rhoifolin potential targets. **(A)** GO cell component enrichment analysis results. **(B)** KEGG pathway enrichment analysis results.

SUPPLEMENTARY FIGURE S2 Kaplan-Meier survival analysis of core genes and their association with overall survival (OS) in lung cancer patients.

SUPPLEMENTARY FIGURE S3 qRT-PCR analysis of EPHB2 mRNA expression in H446 cells treated with Rhoifolin. Mean ± SD n = 3. Statistical significance was determined using p-values, with *p < 0.05, **p < 0.01, and ***p < 0.001 indicating significant differences between groups. “ns” denotes no statistical significance.
