# Correction: Network pharmacology, bioinformatics and in vitro/in vivo validation elucidate the anti-lung cancer activities and potential targets of Rhoifolin

**DOI:** 10.3389/fphar.2026.1788169

**Published:** 2026-02-12

**Authors:** Jing Qian, Wei Cheng, Shuangyan Li, Li Deng, Di Gao, Xue Zhang, Yunhui Zhang

**Affiliations:** 1 Department of Respiratory Medicine, The First People’s Hospital of Yunnan Province, Kunming University of Science and Technology Affiliated Hospital, Kunming, China; 2 Chongqing Key Laboratory of New Drug Screening from Traditional Chinese Medicine, Integrative Science Center of Germplasm Creation in Western China (Chongqing) Science City and Southwest University, SWU-TAAHC Medicinal Plant Joint R&D Centre, College of Pharmaceutical Sciences, Southwest University, Chongqing, China; 3 Department of Oncology, 920th Hospital of the Joint Logistics Support Force of PLA, Kunming, China; 4 Department of Pathology, The First People’s Hospital of Yunnan Province, Kunming University of Science and Technology Affiliated Hospital, Kunming, China; 5 College of Foreign Languages, Qingdao City University, Qingdao, China

**Keywords:** EPHB2, lung cancer, molecular dynamics simulations, network pharmacology, Rhoifolin (ROF)

There was a mistake in Figures 2, 3, 6 as published. In Figures 2F,G, 3D, 6A,D, the compound name “Rhoifolin” was incorrectly spelled as “Phoifolin” in the bar graph for cell lines H358 and H1299. In Figure 2B, the legend label for the “48 h” time point was incorrectly written as “48 4”. In Figure 3D, the asterisks indicating statistical significance on the bar graph were omitted. The corrected Figures 2, 3, 6 appear below.

**FIGURE 2:**
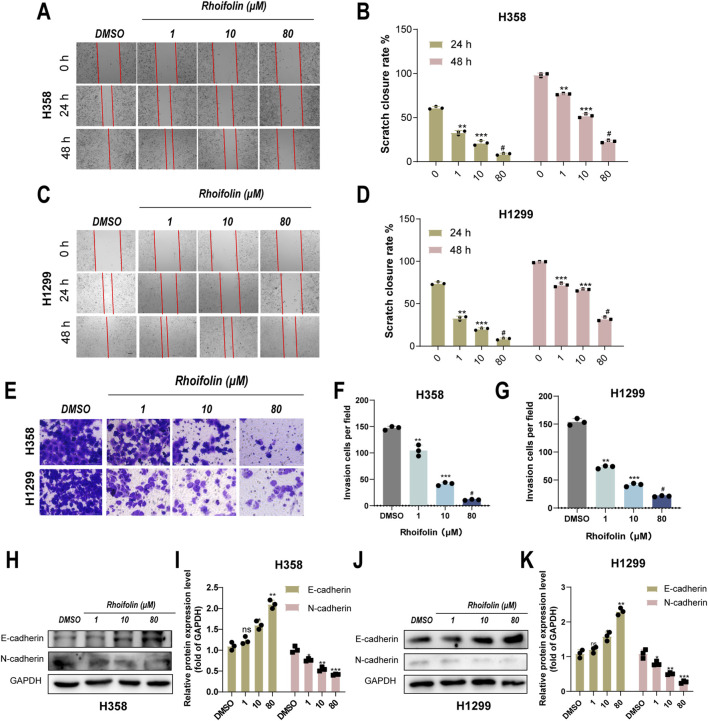
Effect of Rhoifolin on lung cancer cell migration and invasion. **(A–D)** Scratch assay showing the effect of Rhoifolin on the migration of H358 and H1299 cells. **(E–G)** Transwell invasion assays showing the effect of Rhoifolin on the invasion ability of H358 and H1299 cells. **(H–K)** Western blot analysis of EMT-related markers E-cadherin and N-cadherin in H358 and H1299 cells treated with Rhoifolin. Statistical significance was determined using p-values, with *p < 0.05, **p < 0.01, and ***p < 0.001 indicating significant differences between groups. “ns” denotes no statistical significance.

**FIGURE 3:**
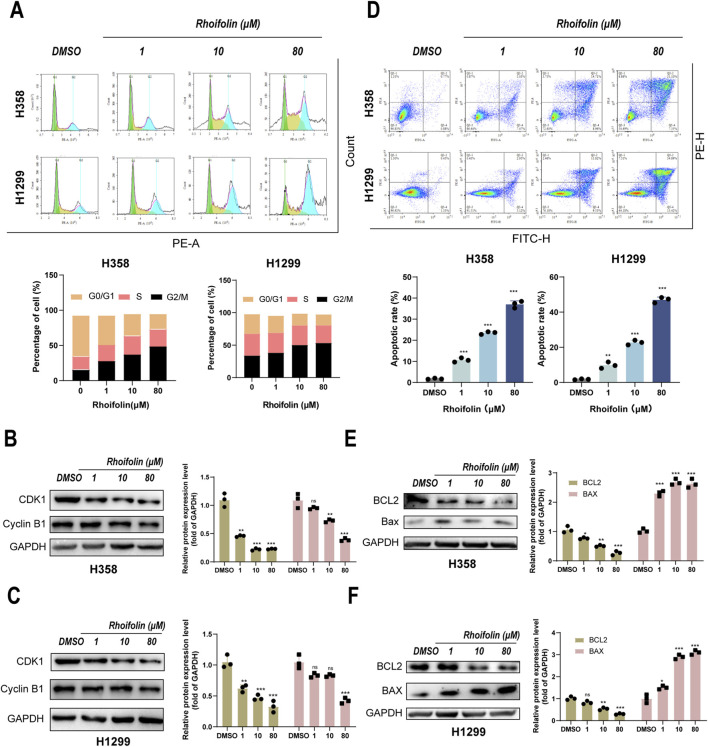
Effect of Rhoifolin on lung cancer cell cycle and apoptosis. **(A)** Flow cytometry analysis showing the effect of Rhoifolin on the cell cycle distribution of H358 and H1299 cells. **(B,C)** Western blot analysis of cell cycle proteins (CDK1,Cyclin B1) in the Rhoifolin-treated H358 and H1299 cells. **(D)** Flow cytometry analysis of apoptosis in H358 and H1299 cells treated with Rhoifolin. **(E,F)** Western blot analysis of apoptotic regulatory proteins (Bax and Bcl-2) in Rhoifolin-treated H358 and H1299 cells. Statistical significance was determined using p-values, with *p < 0.05, **p < 0.01, and ***p < 0.001 indicating significant differences between groups. “ns” denotes no statistical significance.

**FIGURE 6:**
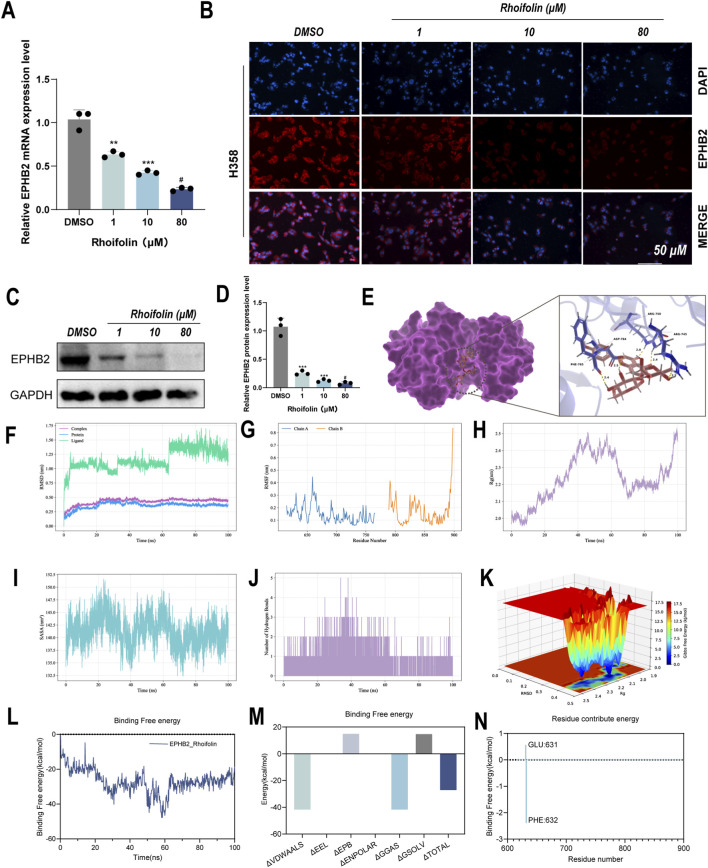
Verification of Rhoifolin potential targeting EPHB2. **(A)** qRT-PCR analysis of EPHB2 mRNA expression in H358 cells treated with Rhoifolin. **(B)** Immunofluorescence observation of EPHB2 protein localization and expression. **(C,D)** Western blot analysis of EPHB2 protein expression in H358 cells treated with Rhoifolin. **(E)** Molecular docking simulation of Rhoifolin binding to EPHB2. **(F)** RMSD analysis of the protein-ligand complex during the simulation. **(G)** RMSF analysis of the protein-ligand complex during the simulation. **(H)** Radius of gyration (Rg) analysis of the protein-ligand complex. **(I)** SASA analysis of the protein-ligand complex. **(J)** Hydrogen bond analysis of the protein-ligand complex during the simulation. **(K)** Free energy landscape of the protein-ligand complex during the simulation. **(L)** Dynamic change in binding free energy of the Rhoifolin-EPHB2 complex during 100 ns simulation. **(M)** Total binding free energy of the Rhoifolin-EPHB2 complex. **(N)** Free energy decomposition analysis of key residues in the Rhoifolin-EPHB2 complex.

The original article has been updated.

