# Supplementary figures and images for: Network pharmacology, bioinformatics and in vitro/in vivo validation elucidate the anti-lung cancer activities and potential targets of Rhoifolin
Source: Front Pharmacol. 2026 Jan 14;16:1727729. doi: 10.3389/fphar.2025.1727729 (PMC12847355; doi:10.3389/fphar.2025.1727729)

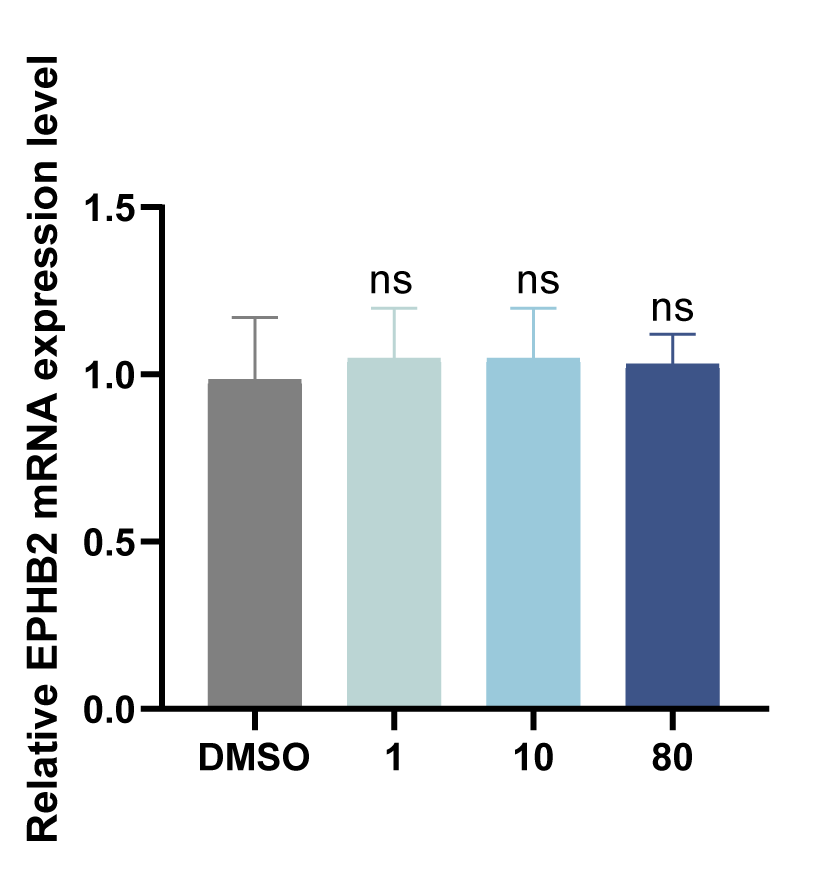

Supplement: Supplementary file 1 [file Image3.tif]

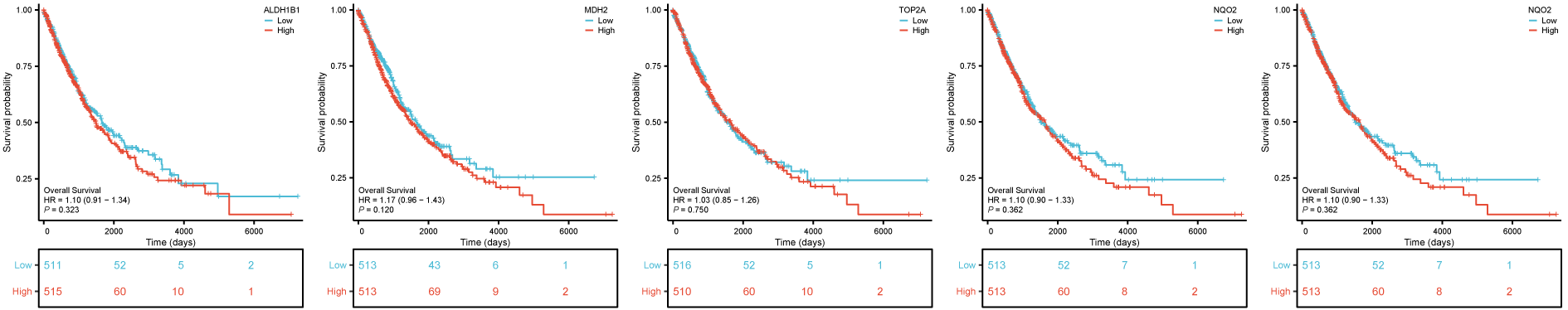

Supplement: Supplementary file 2 [file Image2.tif]

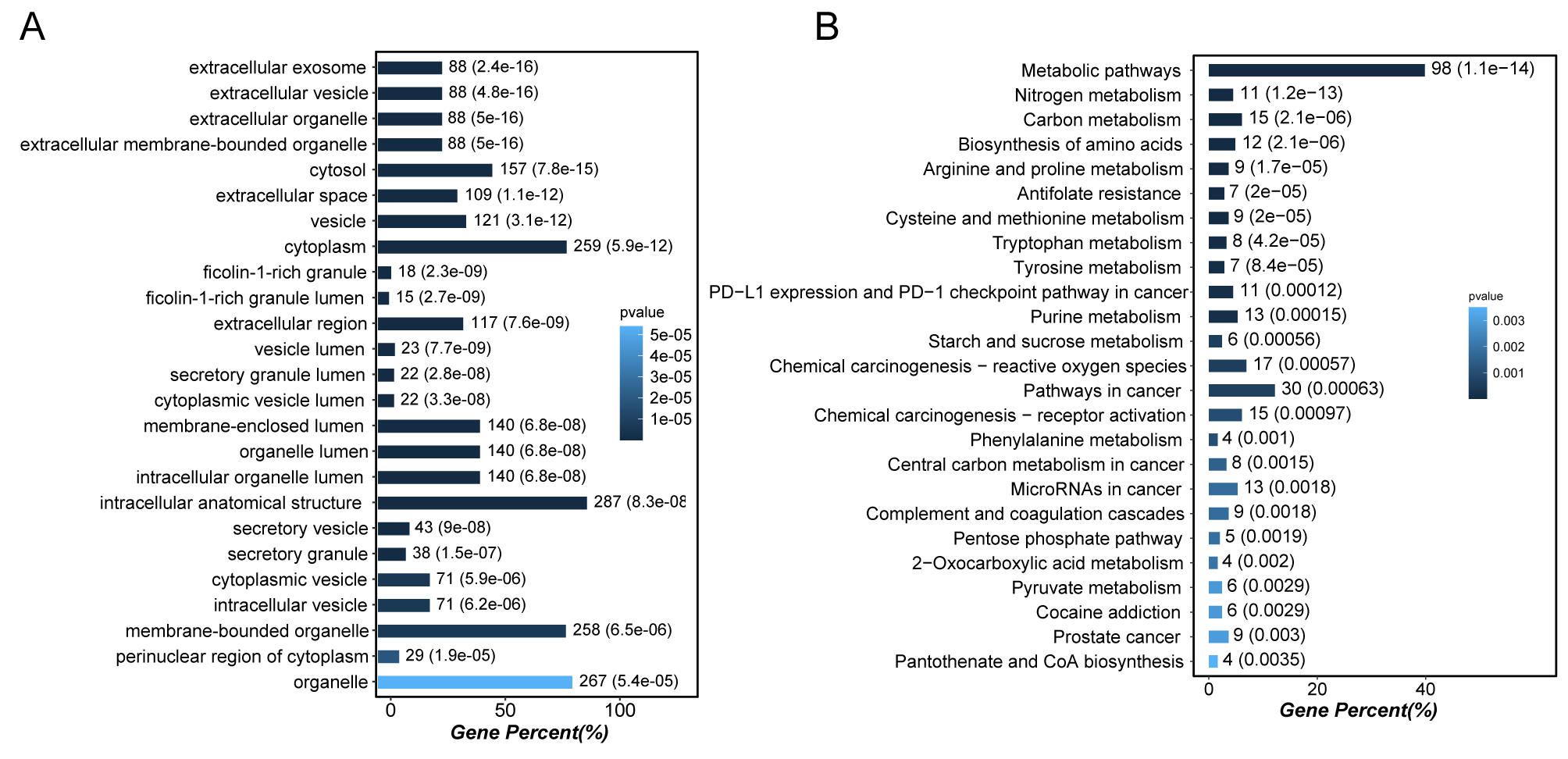

Supplement: Supplementary file 3 [file Image1.tif]
